# Screening of *Strongyloides stercoralis* infection in high-risk patients in Khuzestan Province, Southwestern Iran

**DOI:** 10.1186/s13071-020-04549-6

**Published:** 2021-01-09

**Authors:** Alireza Ashiri, Abdollah Rafiei, Molouk Beiromvand, Abdollah Khanzadeh, Arash Alghasi

**Affiliations:** 1 Infectious and Tropical Diseases Research Center, Health Research Institute, Ahvaz Jundishapur University of Medical Sciences, Ahvaz, Khuzestan Iran; 2 Department of Parasitology, School of Medicine, Ahvaz Jundishapur University of Medical Sciences, Ahvaz, Khuzestan Iran; 3 Abadan School of Medical Sciences, Abadan, Khuzestan Iran; 4 Thalassemia and Hemoglobinopathy Research Center, Health Research Institute, Ahvaz Jundishapur University of Medical Sciences, Ahvaz, Khuzestan Iran

**Keywords:** *Strongyloides stercoralis*, Strongyloidiasis, Risk factors, High-risk patients, Corticosteroid, Iran

## Abstract

**Background:**

Strongyloidiasis, one of the neglected tropical diseases (NTDs), can be fatal in immunocompromised patients. Available data on *Strongyloides stercoralis* infection in high-risk patients in Iran are limited. The aim of the present study was to determine the prevalence of *S. stercoralis* infection and associated risk factors among high-risk patients as well as to evaluate the sensitivity of the diagnostic tests used in the diagnose of *S. stercoralis* infection.

**Methods:**

This cross-sectional study was performed from 2019 to 2020 among 300 high-risk patients in Khuzestan Province, southwestern Iran. Patients with autoimmune diseases, uncontrolled diabetes, HIV/AIDS, cancer, organ transplant, hematological malignancy, asthma and chronic obstructive pulmonary disease (COPD) were examined using direct smear examination, formalin-ether concentration, Baermann funnel technique, agar plate culture, and ELISA test. Since agar plate culture was considered the reference diagnostic test, culture-positive samples were confirmed by PCR amplification and the sequencing of the nuclear *18S* rDNA (*SSU*) hypervariable region (HVRIV) of the parasite.

**Results:**

The prevalence of *S. stercoralis* infection was 1%, 1.3%, 2%, 2.7%, and 8.7% using direct smear examination, formalin-ether concentration, Baermann funnel technique, agar plate culture, and ELISA test, respectively. All culture-positive samples were confirmed by *SSU*-PCR. According to the results, the most sensitive test was ELISA, with 100% sensitivity, followed by the Baermann funnel technique with the sensitivity of 75%. Direct smear examination, formalin-ether concentration technique, and Baermann funnel technique had the highest PPV (100%) while the ELISA test had the highest NPV (100%). Significant eosinophilia was observed in the patients whose culture test was positive (7/8; *P* < 0.05). In the present study, the majority of the positive cases by the agar plate culture had a history of prolonged exposure to soil and of asthma and COPD and were > 60 years old.

**Conclusions:**

Given that the ELISA test had the highest NPV, the screening of all high-risk patients for *S. stercoralis* infection in endemic areas is recommended prior to starting corticosteroid therapy with the ELISA test. The results indicate the importance of paying attention to patients with unknown eosinophilia in endemic areas. Ivermectin should be available to strongyloidiasis patients in the endemic areas.
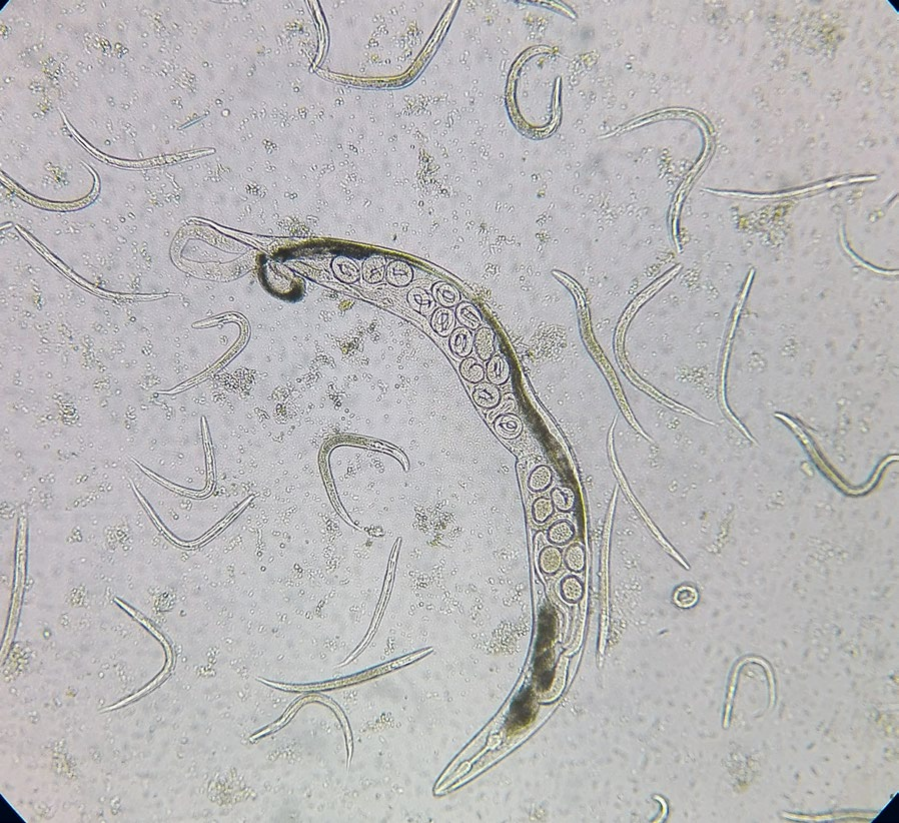

## Background

Strongyloidiasis is a soil-transmitted helminthiasis (STH), caused mainly by the species *Strongyloides stercoralis.* This intestinal nematode, with a prevalence of nearly 30−100 million people worldwide, is one of the neglected tropical diseases (NTD) [1, 2]. *S. stercoralis* infection generally occurs in tropical and subtropical countries, particularly in areas with warm and humid climates [3, 4]. Although this nematode has a complex life-cycle, the most frequent infection route is percutaneous entry of the filariform larvae [4]. Clinical manifestations of strongyloidiasis vary from asymptomatic or mild gastrointestinal and/or cutaneous symptoms in healthy people [5] to severe clinical symptoms in immunocompromised patients, which lead to hyperinfection syndrome or disseminated strongyloidiasis [6]. Various factors, including corticosteroid drugs, human T-cell lymphotropic virus type 1 (HTLV-1) infection, malnutrition, diabetes mellitus (DM), chronic obstructive pulmonary disease (COPD) [7], transplant, and human immunodeficiency virus (HIV) infection impair immune responses and put the patient at risk for the severe strongyloidiasis [1].

Several parasitological and serological tests are available to diagnose *S. stercoralis* infection. Among those, agar plate culture is currently regarded as the most sensitive parasitological technique. However, this technique has limitations due to the need for fresh stool and might be time-consuming [8]. Among the serological tests, enzyme-linked immunosorbent assay (ELISA) is increasingly being used to overcome the limitations of parasitological techniques. Nevertheless, this method also has some limitations, including cross reaction with other helminthic infections and difficulty in distinguishing between current and past *S. stercoralis* infection [5].

Iran, as a tropical and subtropical region, is an endemic area for *S. stercoralis*; however, most reports come from the northern and southern parts of the country [9–11]. In a study conducted by Rafiei et al. [9], the seroprevalence of *S. stercoralis* was reported to be 14.4% in immunocompromised patients from southwestern Iran. Since many strongyloidiasis cases are asymptomatic and conventional parasitological examinations are not sufficiently sensitive, *S. stercoralis* infection is frequently under-diagnosed [7]. Nonetheless, the potential of uncontrolled multiplication and life-threatening dissemination of *S. stercoralis* larvae in immunocompromised patients can lead to mortality rates of 85% [12]. Despite the importance of the early detection of *S. stercoralis* in immunocompromised patients, very few studies have investigated strongyloidiasis in high-risk groups, particularly patients receiving immunosuppressive drugs in Iran [9, 13–15]. In addition, available data on strongyloidiasis in Iranian high-risk patients are limited to a few studies conducted with only one or two diagnostic methods. The main aim of the present study was to assess the prevalence of *S. stercoralis* in high-risk patients in Abadan and Ahvaz Counties, Khuzestan Province, southwestern Iran, and to evaluate the sensitivity of the diagnostic tests used in diagnosing *S. stercoralis* infection.

## Methods

### Study area

Khuzestan Province (31.3273° N, 48.6940° E), 1 of the 31 provinces of Iran, is located in the southwest of the country, bordering Iraq and the Persian Gulf. Most of the province has a mild winter and very hot summer. However, some parts of Khuzestan Province have humid summers. In the northern parts of the province wheat, barley, rice, and sugar cane are cultivated while in southern parts, such as Abadan and Khoramshahr Counties, produce date palms.

### Study population, study design and clinical assessment

**Fig. 1:**
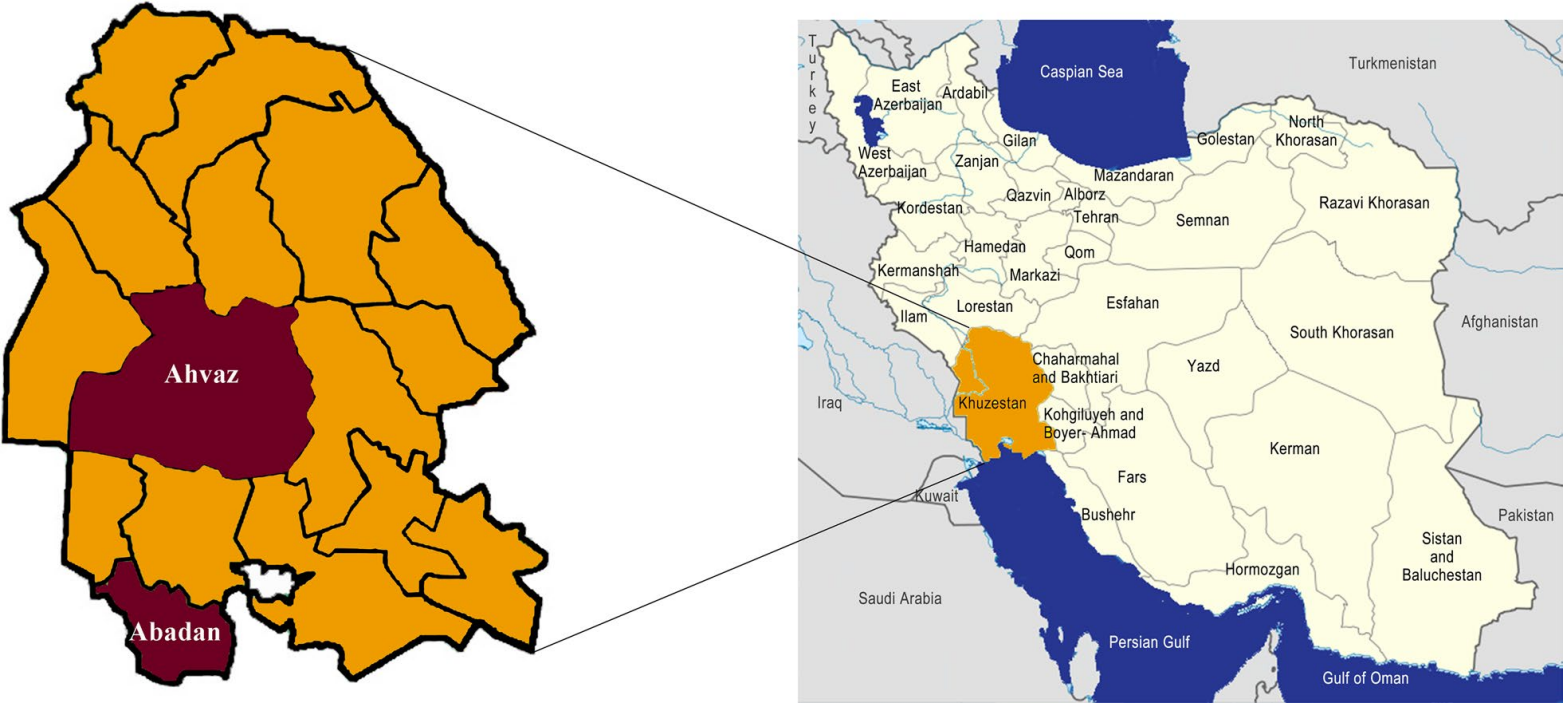
Map of Iran. Khuzestan Province, highlighted in dark orange, based on the map of Iran in [16]. Map of Khuzestan Province with studied counties highlighted in dark red

This cross-sectional study was designed to evaluate the prevalence of *S. stercoralis* among the patients referred to 17 Shahrivar (Abadan) and Shahid Baghaee (Ahvaz) hospitals from June 2019 to March 2020 (Fig. 1) [16]. The selected counties were chosen based on previous studies [9, 17] and hospital reports. The sample size was determined using the prevalence rate of 14.4% from a previous study [9]. The calculated sample size was 296 patients; thus, 300 patients were enrolled in the study. Simple random sampling of the patients was carried out according to the number of high-risk patients referred to each hospital. Inclusion criteria were high-risk groups, including patients with autoimmune diseases, rheumatoid arthritis, uncontrolled diabetes (fasting blood sugar level > 300 mg/dl or HbA1c > 9), HIV/AIDS, and cancer; organ transplanted cases; patients receiving corticosteroids; malnourished patients; and patients with asthma and chronic obstructive pulmonary disease (COPD). Children < 5 years old, patients who had undergone anti-helminth therapy in the past 6 months, patients living outside the study area, and those who did not provide a stool specimen were excluded from the study.

Socio-demographic data (age, gender, and residency area) and clinical characteristics of all patients were collected using a standardized questionnaire. A clinical assessment was conducted by an internal medicine physician and an oncologist at 17 Shahrivar (Abadan) and Shahid Baghaee (Ahvaz) hospitals, respectively.

### Laboratory investigation procedures

Clean plastic flasks were provided to the patients or their companions, and fecal samples were collected the following day. Two-milliliter venous blood samples were obtained from each patient for the ELISA test. After rapid transfer to the laboratory, the sera were stored at − 20 °C for the ELISA test, and the fecal samples were processed immediately for further examinations.

#### Direct smear examination

The fecal samples were examined microscopically to detect *S. stercoralis* larvae at 100× and 400× magnifications using the saline/Lugol iodine staining method. To increase the sensitivity, three smears of each sample were examined.

#### Formalin-ether concentration technique

Fecal samples were analyzed by the formalin-ether concentration technique as described previously [18]. Approximately 1 g of each specimen was suspended in 10 ml saline (0.9%) and was passed through a four-layer gauze. The suspension was centrifuged at 700×*g* for 5 min at room temperature. After discarding the supernatant, 7 ml formalin (10%) and 3 ml diethyl ether were added to the sediment. The suspension was shaken vigorously for 1 min and centrifuged at 700×*g* for 5 min. The top three layers were removed, and the sediment was examined microscopically at 100× and 400× magnifications [18].

#### Baermann funnel technique

Approximately 4–5 g fresh fecal samples were placed on the center of a four-layer gauze, which was closed using a rubber band. The specimen was immersed in a funnel filled with lukewarm water; its bottom was blocked. This was left for 16–18 h at room temperature. The collected suspension was centrifuged at 500×*g* for 2 min, and then the sediment was analyzed using normal saline at 100× and 400× magnifications.

#### Agar plate culture

For the agar plate culture, approximately 3 g of fresh fecal sample was placed on the center of a nutrient agar plate. The plate was sealed with cellulose tape and placed into a box at room temperature (25–30 °C) for 7 days. On days 2 through 7, the surfaces of plates were washed with normal saline solution and centrifuged at 1500 rpm for 5 min to collect larval and adult forms of *S. stercoralis.* To discriminate *S. stercoralis* and hookworm larvae morphologically, the sediment was examined under 400× microscopic magnification. Based on previous studies, the agar plate culture method was considered the most efficient parasitological technique.

#### Enzyme-linked immunosorbent assay (ELISA)

An ELISA test was conducted using an IgG ELISA (NovaTec Immunodiagnostica GmbH, Dietzenbach, Germany) kit using recombinant immunodiagnostic antigen (NIE) according to the manufacturer's instructions. The absorbance values were measured at 450 nm using a Dynex DS2® automated ELISA reader. The results were calculated using NovaTec units (NTU). The cut-off was determined to be 10 NTU. Values > 11 NTU were reported as positive; those between 9 and 11 NTU and < 9 NTU were reported as equivocal and negative, respectively.

#### Molecular analysis

DNA extraction was performed on agar plate culture-positive samples as described previously [19]. The collected larval and adult forms were frozen for 5 min in liquid nitrogen and thawed for 5 min in a boiling water bath. Freezing and thawing step was repeated five times. The lysate was stored at − 20 °C until use.

The confirmation of the *S. stercoralis* infection was performed by a PCR protocol to amplify a 712-bp fragment of the nuclear *18S* rDNA (*SSU*) hypervariable region (HVRIV) of the parasite as described before [19]. The PCR was carried out in a 20-μl reaction volume containing 10 μl of 2× Master Mix RED (Ampliqon-Biomol, Hamburg, Germany), 0.5 μl of each forward and reverse primer, 7 μl nuclease-free water, and 2 μl template DNA. The 712 -p fragment was amplified using forward primer 18SP4F (5′-GCGAAAGCATTTGCCAA-3′) and reverse primer 18SPCR (5′-ACGGCCGGTGTGTAC-3′) as described by Hasegawa et al. [20]. The PCR cycling was as follows: an initial denaturation at 95 °C for 30 s, followed by 35 cycles of 95 °C for 20 subtypes (denaturation), 55 °C for 15 s (annealing), 68 °C for 90 s (extension), and a final extension of 68 °C for 5 min. The PCR product was used for sequencing using primer ZS6269 (5′-GTGGTGCATGGCCGTTC-3′) at Pishgam Biotech Co. (Tehran, Iran) [21]. The obtained sequences were assembled using Chromas version 2.1 and were compared with sequences previously deposited at the National Center for Biotechnology Information (NCBI) using the BLAST tool (http://www.ncbi.nlm.nih.gov/blast).

### Statistical analysis

Data analysis was conducted using SPSS 22 software (SPSS Inc., Chicago, IL, USA). To compare proportions, the chi-square test was used. The agar plate culture was compared with other coproparasitological and ELISA methods using chi-square and Fisher’s exact tests. The receiver-operating characteristic (ROC) curve used to evaluate the coproparasitological and ELISA test was compared to the agar plate culture for diagnosis of *S. stercoralis* larva. Moreover, the sensitivity, specificity, positive predictive value (PPV), and negative predictive value (PNV) of the diagnostic methods were estimated. The Kappa test was used to assess concordance between the agar plate culture test and other diagnostic tests.

## Results

### Patient characteristics

Of the 300 patients participating in the study, 27.3% had a history of uncontrolled diabetes, 34.0% had autoimmune diseases (17.7%) or asthma and chronic obstructive pulmonary disease (COPD) (16.3%) and were receiving steroids, 26.3% had malignant diseases (cancer and leukemia), and 12.4% had other diseases (AIDS, solid organ transplantation, and malnutrition). Regarding gender, 144 (48.0%) were male and 156 (52.0%) female. The age range of the patients was 5−93 (mean 54.71; SD: 18.1) years. Of these, 41.7% (125/300) were in > 60 years old. Regarding the place of residence, 67.3% (202/300) were from urban and 32.7% (98/300) from rural areas. According to Table 1, 43.0%, 10.7%, and 7.3% of the patients were housewives, farmers, and fishermen, respectively. Gastrointestinal and pulmonary symptoms were reported by 32.7% and 12.7%, respectively. In 9.0% of the patients, both gastrointestinal and pulmonary symptoms were observed. At the time of referral, 51 (17.0%) of the patients had diarrhea (Table 1). Data on eosinophils was obtained by examining the patients' files. Of the 175 patients for whom CBC tests were performed, 46 (26.3%) had eosinophil counts > 10.0%. Of these, the highest percentage (64.0%) was of the patients treated with steroids.

**Table 1:** Socio-demographic and clinical characteristics of the patients according to the underlying disease

Characteristics	All patients No. = 300	Uncontrolled diabetes No. = 82	Malignant diseases No. = 79	Steroid therapy No. = 102	Other diseases No. = 37
	*n* (%)	*n* (%)	*n* (%)	*n* (%)	*n* (%)
Age (years)					
< 20	16 (5.3)	1 (6.3)	13 (81.3)	2 (12.5)	0 (0)
20−40	45 (15.0)	3 (6.7)	17 (37.8)	19 (42.2)	6 (13.3)
41−60	114 (38.0)	40 (35.1)	29 (25.4)	28 (24.6)	17 (14.9)
60+	125 (41.7)	38 (30.4)	20 (16.0)	53 (42.4)	14 (11.2)
Gender					
Male	144 (48.0)	32 (22.2)	50 (34.7)	37 (25.7)	25 (17.4)
Female	156 (52.0)	50 (32.1)	29 (18.6)	65 (41.7)	12 (7.7)
Living area					
Rural	98 (32.7)	34 (34.7)	24 (24.5)	26 (26.5)	14 (14.3)
Urban	202 (67.3)	48 (23.8)	55 (27.2)	76 (37.6)	23 (11.4)
Clinical symptoms					
Gastrointestinal	98 (32.7)	41 (41.8)	24 (24.5)	13 (13.3)	20 (20.4)
Pulmonary	38 (12.7)	4 (10.5)	3 (7.9)	30 (79.0)	1 (2.6)
Gastrointestinal/ pulmonary	27 (9.0)	0 (0.0)	4 (14.8)	22 (81.5)	1 (3.7)
Asymptomatic	137 (45.6)	37 (27.0)	48 (35.1)	37 (27.0)	15 (10.9)
Diarrhea					
Yes	51 (17.0)	17 (33.3)	11 (21.6)	11 (21.6)	12 (23.5)
No	249 (83.0)	65 (26.1)	68 (27.3)	91 (36.6)	25 (10.0)
Soil exposure					
Low	221 (73.7)	58 (26.3)	57 (25.8)	79 (35.7)	27 (12.2)
High	79 (26.3)	24 (30.4)	22 (27.8)	23 (29.1)	10 (12.7)

#### *Strongyloides stercoralis* prevalence, ROC curves, predictive values, and concordance

**Fig. 2:**
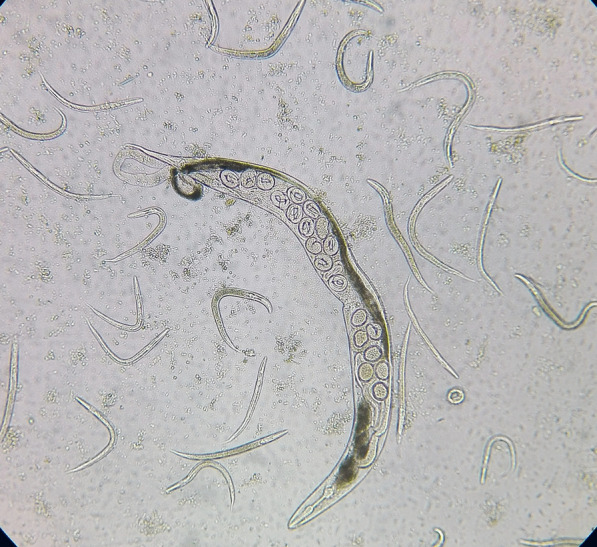
Adult female *Strongyloides stercoralis* worm, rhabditiform larvae, and ova collected from an agar plate culture of an infected patient examined in this study

**Fig. 3:**
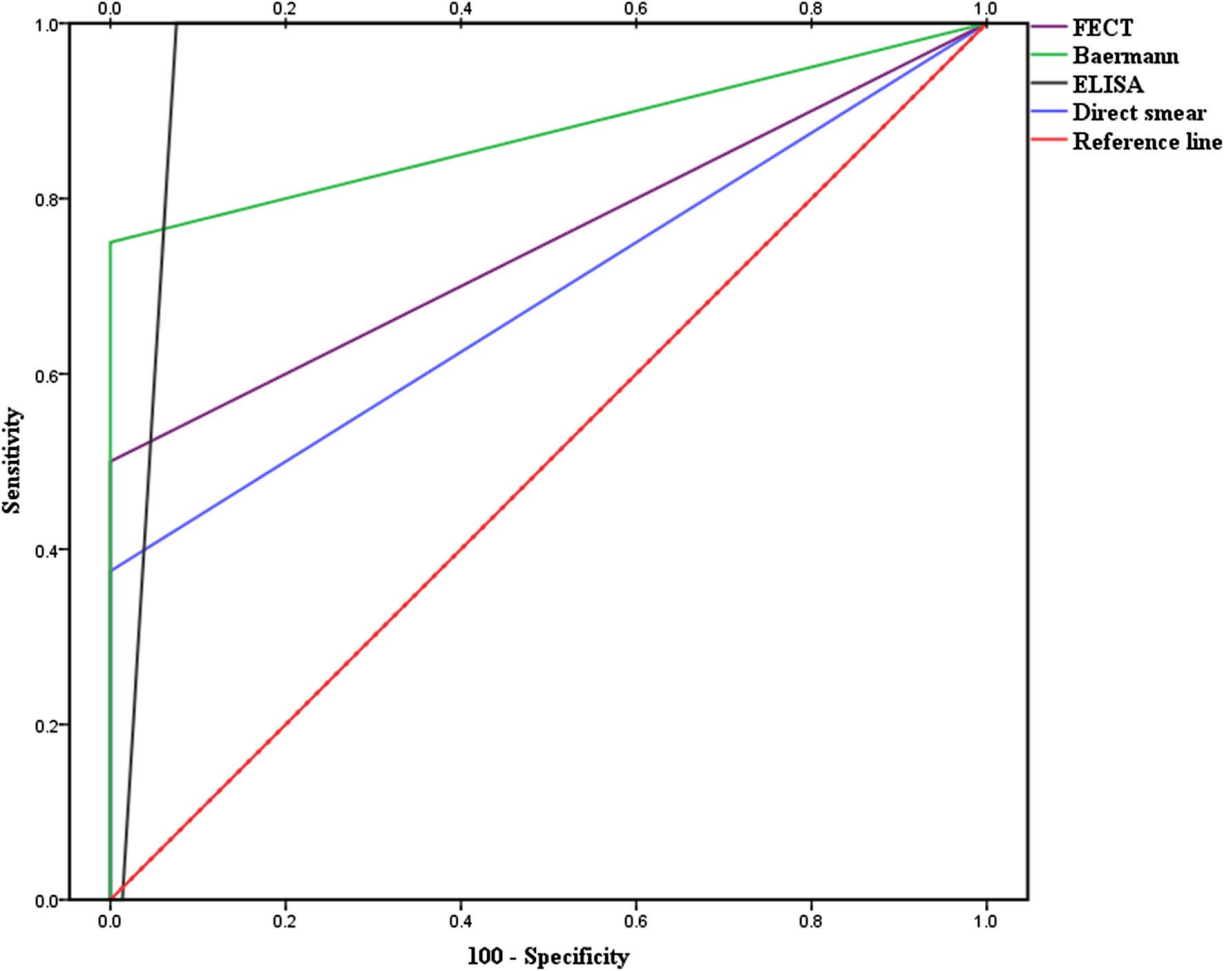
The ROC curves show the comparison between the agar plate culture and other tests used. *FECT* formalin-ether concentration technique. The ELISA ROC had an AUC (area under the curve) of 0.955 with 100% sensitivity, the Baermann funnel technique had an AUC of 0.875 with 70% sensitivity, the formalin-ether concentration ROC had an AUC of 0.750 with 50% sensitivity, and the direct smear examination ROC had an AUC of 0.688 with 37.5% sensitivity

**Fig. 4:**
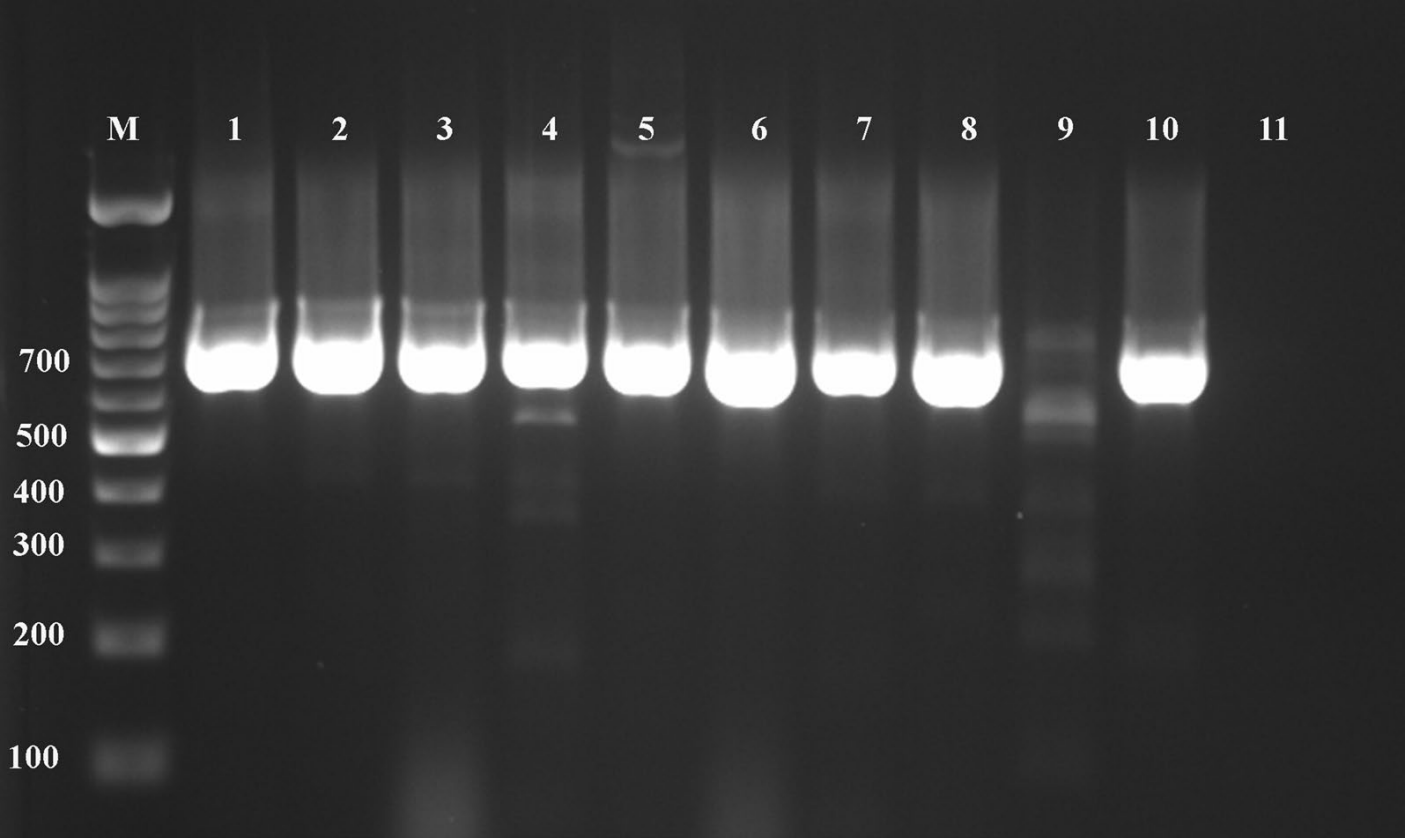
PCR amplification of a 712-bp fragment of the nuclear 18S rDNA (*SSU*) hypervariable region (HVRIV) of *Strongyloides stercoralis* from larvae in feces of high-risk patients from Khuzestan Province, Iran. Lane M: 100-bp DNA ladder RTU (CinnaGen, Iran); lanes 1–8: positive samples; lanes 9, 10: positive controls; lane 11: negative control

The observed prevalence of *S. stercoralis* was 1% (3/300; 95% confidence interval: 1% to 1.02%; *P* > 0.05) using direct smear examination. Using the formalin-ether concentration, Baermann funnel technique, agar plate culture, and ELISA, the prevalence was 1.3% (4/300; *P* > 0.05), 2% (6/300; *P* > 0.05), 2.7% (8/300; *P* > 0.05), and 8.7% (26/300; *P* > 0.05), respectively (Fig. 2; Table 2). All patients who were positive with lower sensitivity methods were also positive with higher sensitivity methods, and none of the parasitology positive cases were negative according to ELISA. The direct smear examination ROC had an AUC (area under the curve) of 0.688 with 37.5% sensitivity compared to the agar plate culture. Furthermore, when the other tests were compared to the agar plate culture, the formalin-ether concentration ROC had an AUC of 0.750 with 50% sensitivity, the Baermann funnel technique had an AUC of 0.875 with 70% sensitivity, and the ELISA test had an AUC of 0.955 with 100% sensitivity and 93.8% specificity (Fig. 3). Compared with the agar plate culture, the ELISA test was found to be the most sensitive test, with a sensitivity of 100%, followed by the Baermann funnel technique with 75% sensitivity. According to the data collected in Table 3, the direct smear examination, formalin-ether concentration technique, and Baermann funnel technique had the highest PPV (100%) while the ELISA test had the highest NPV (100%). The Baermann funnel technique showed the highest agreement with the agar plate culture (Kappa; *κ* = 0.854) followed by the formalin-ether concentration (*κ* = 0.661). Data summarized in Table 4 indicate that the occupation of patients, type of underlying disease, clinical symptoms, eosinophilia, soil exposure, and diagnostic methods were significantly associated with the presence of *S. stercoralis* larvae according to the agar plate culture (*P* < 0.05). The obtained culture results indicated that no significant differences were observed among gender, residency, and infection (*P* > 0.05). However, of the eight culture-positive cases, seven patients were from Abadan County. A marginal trend toward significance was found between the age groups and *S. stercoralis* infection. Of the eight culture-positive patients, seven were > 60 years old (*P* = 0.052). Among the clinical factors, significant eosinophilia was observed in the patients whose culture test were positive (7/8; *P* < 0.05). Moreover, of the 26 ELISA-positive patients, 17 (65.4%; *P* < 0.05) had eosinophilia. All culture-positive samples were confirmed by PCR (Fig. 4). Of the eight culture-positive patients, two were positive 6 and 8 months after treatment, respectively, according to the agar plate culture.

**Table 2:** Frequency of *Strongyloides stercoralis* larvae in stool samples of the 300 patients studied, according to coproparasitological and serological methods

Techniques	Uncontrolled diabetes No. = 82	Malignant diseases No. = 79	Steroid therapy No. = 102	Other diseases No. = 37	*P*
	*n* (%)	*n* (%)	*n* (%)	*n* (%)	
Direct smear examination					
Positive	1 (33.3)	0 (0.0)	2 (66.7)	0 (0.0)	0.539
Negative	81 (27.3)	79 (26.6)	100 (33.7)	37 (12.4)	
Formalin-ether concentration technique					
Positive	1 (25.0)	0 (0.0)	3 (75.0)	0 (0.0)	0.311
Negative	81 (27.4)	79 (26.7)	99 (33.4)	37 (12.5)	
Baermann funnel technique					
Positive	1 (16.7)	1 (16.7)	4 (66.7)	0 (0.0)	0.369
Negative	81 (27.6)	78 (26.5)	98 (33.3)	37 (12.6)	
Agar plate culture					
Positive	1 (12.5)	1 (12.5)	6 (75.0)	0 (0.0)	0.096
Negative	81 (27.7)	78 (26.7)	96 (32.9)	37 (12.7)	
Enzyme-linked immunosorbent assay					
Positive	11 (42.3)	3 (11.5)	9 (34.7)	3 (11.5)	0.287
Equivocal	1 (25.0)	2 (50.0)	0 (0.0)	1 (25.0)	
Negative	70 (25.9)	74 (27.4)	93 (34.5)	33 (12.2)

**Table 3:** Sensitivity, specificity, positive predictive value, negative predictive value, and concordance between the agar plate culture and other diagnostic tests used in this study

Diagnostic tests	Sensitivity%	Specificity%	PPV%	NPV%	Concordance
DSE	37.5	100	100	98.3	0.539
FECT	50	100	100	98.6	0.661
BFT	75	100	100	99.3	0.854
ELISA	100	93.8	30.8	100	0.448

*DSE* direct smear examination, *FECT* formalin-ether concentration technique, *BFT* Baermann funnel technique; *ELISA* enzyme-linked immunosorbent assay, *PPV* positive predictive value; *NPV* negative predictive value

**Table 4:** Socio-demographic and clinical characteristics of the 300 patients associated with *Strongyloides stercoralis* infection, according to the agar plate culture

Characteristics	Positive (%)	Negative (%)	*P*
Gender			
Male	6 (4.1)	139 (95.9)	0.162
Female	2 (1.3)	153 (98.7)	
Age (years)			
< 20	0 (0.0)	16 (100.0)	0.052
20−40	1 (2.2)	44 (97.8)	
41−60	0 (0.0)	114 (100.0)	
60+	7 (5.6)	118 (94.4)	
Living area			
Rural	4 (4.1)	94 (95.9)	0.445
Urban	4 (2.0)	198 (98.0)	
Occupation			
Housewife	2 (1.6)	127 (98.4)	0.002
Farmer	4 (12.5)	28 (87.5)	
Worker	2 (7.7)	24 (92.3)	
Fisherman	0 (0.0)	22 (100.0)	
Student	0 (0.0)	13 (100.0)	
Other	0 (0.0)	78 (100.0)	
Diseases			
Cancer	0 (0.0)	46 (100.0)	0.032
Uncontrolled diabetes	1 (1.2)	81 (98.8)	
Autoimmune diseases	1 (1.9)	52 (98.1)	
Hematological malignancy	1 (3.0)	32 (97.0)	
Asthma and COPD	5 (10.2)	44 (89.8)	
Transplant	0 (0.0)	9 (100.0)	
Other	0 (0.0)	28 (100.0)	
Clinical symptoms			
Gastrointestinal	1 (1.0)	97 (99.0)	0.017
Pulmonary	2 (5.3)	36 (94.7)	
Gastrointestinal/pulmonary	3 (11.1)	24 (88.9)	
Asymptomatic	2 (1.5)	135 (98.5)	
Eosinophilia			
Yes	7 (15.2)	39 (84.4)	0.001
No	1 (0.8)	128 (99.2)	
Soil exposure			
Low	2 (0.9)	219 (99.1)	0.002
High	6 (7.6)	73 (92.4)	
Diagnostic method			
DSE	3 (37.5)	5 (62.5)	0.001
FECT	4 (50.0)	4 (50.0)	
BFT	6 (75.0)	2 (25.0)	
ELISA	8 (100.0)	0 (0.0)

## Discussion

In this study, the prevalence of *S. stercoralis* with the agar plate culture was lower than that using the ELISA test but was higher compared to the formalin-ether concentration technique, Baermann funnel technique, and the direct smear examination. Direct smear examination had the lowest sensitivity in diagnosing *S. stercoralis* (37.5%). These findings match those observed in previous studies [3, 22, 23]. In up to 70% of the strongyloidiasis cases, direct smear examination using a single stool test fails to diagnose the infection [24], but serial stool examinations can increase the chance of diagnosing. According to a study conducted by Khieu et al. [25], the prevalence of *S. stercoralis* infection increased from 15.9 to 21.6% by analyzing three stool samples. In chronic strongyloidiasis, the sensitivity of a single stool examination in detecting *S. stercoralis* in symptomatic patients is approximately 50% while in asymptomatic patients it is probably less [24, 26]. Moreover, the low parasite load and intermittent excretion of *Strongyloides* larvae make it difficult to detect microscopically [27]. It should be noted that sampling on consecutive days, especially in patients with underlying diseases, is very difficult and impractical. Thus, more sensitive methods should be used to increase the chance of strongyloidiasis detection.

The prevalence observed by the ELISA test (8.7%) was lower than that reported by Rafiei et al. [9] in the same region. Although ELISA was used to overcome the limitations of parasitological methods, the discrepancy observed might be related to the cross reactivity in patients with other STH infections and/or the difference in the sensitivity of the ELISA kits. Another possible explanation for this result might be that we used the NovaLisa *Strongyloides* ELISA Kit (NovaTec Immunodiagnostica, Germany), which uses a recombinant antigen (NIE), while Rafiei et al. used the Bordier-ELISA kit (Bordier Affinity Products SA, Switzerland), which uses somatic antigens.

According to the obtained results (Table 3), the direct smear examination, formalin-ether concentration, and Baermann funnel technique were the tests with the highest specificity but had low sensitivity. However, ROC analysis showed that the ELISA test with an AUC of 0.955 and the sensitivity of 100% was the most sensitive test in diagnosing strongyloidiasis. It seems that both parasitological and serological tests and serial stool examinations should be used to diagnose *Strongyloides* infection, especially for high-risk patients in endemic areas.

Based on the agar plate culture, the prevalence of *S. stercoralis* in males was slightly higher than that in females, but the difference was not significant (Table 4). This may be due to the fact that some men are more exposed to contaminants because of working outside the home. These findings are consistent with those of Luvira et al. [8] who found higher infection rates in males but differ from those of other studies [9, 22]. Regarding the place of residence, we did not find a significant difference between the rural and urban areas (*P* > 0.05). Several studies have reported that living in rural areas and prolonged exposure to contaminated soil may put humans at the risk of *S. stercoralis* infection [3, 28]. In Iran, migration from rural to urban areas has increased in recent years. Therefore, it is possible that some patients who live in the urban areas were infected during their residency in rural areas. In our study, of the eight positive cases according to the agar plate culture, 75% had a history of prolonged exposure to soil. Given the fact that *S. stercoralis* is a soil-transmitted helminth, in endemic areas such as Khuzestan Province, walking barefoot and occupational exposure of some patients to contaminated soil may increase the chance of infection. In our study, a significant association was found between occupation and infection. Farmers with a prevalence of 50% had the highest infection rate. Age-related findings revealed that most cases diagnosed by agar plate culture (87.5%) and ELISA test (57.7%) were patients > 60 years old. These results differ from previous published studies [3, 9], but agree with the findings of other studies [29–31]. These results may be explained by the fact that *S. stercoralis* has a unique life-cycle that allows autoinfection [5]. Therefore, in the elderly population, whose immune systems are weakened [31], parthenogenesis by the adult worm may lead to accelerated autoinfection and clinical presentations [5]. Clinical manifestations such as gastrointestinal and pulmonary symptoms were associated with the agar plate culture results (*P* = 0.017) but not with the ELISA test (*P* = 0.311). The ability of the parasite to replicate might lead to persistence within a host for decades. This discrepancy between ELISA results and clinical symptoms could be attributed to past *S. stercoralis* infections.

Peripheral eosinophilia as high as 75–80% is common during acute infection but in chronic infection is intermittent. In severe strongyloidiasis and immunocompromised patients, eosinophilia is frequently absent [32]. In our study, 87.5% of the culture-positive cases had an eosinophil count > 10.0%. The significant association between infection and eosinophilia (*P* = 0.001) might be related to the fact that *S. stercoralis* female worms live within the submucosa of the gut, and thus the eosinophilic response may occur at a higher level compared to other chronic intestinal parasitic infections [7]. In a study conducted by Ashrafi et al. [33] in Gilan Province, Northern Iran, out of 150 patients with undiagnosed eosinophilia, 42% were diagnosed with strongyloidiasis. In the present study, of the seven positive patients who had eosinophilia, five had a history of asthma and COPD. Therefore, this finding might be related to their underlying diseases.

Statistical analysis showed that the Baermann funnel technique had the highest agreement with the agar plate culture (0.854), so that it was detected in 75% of the culture-positive cases. This finding is in agreement with that of Hailegebriel et al. [22], who found that 75% of *S. stercoralis* infection was diagnosed by either the agar plate culture or Baermann funnel technique. The sensitivity of the agar plate culture compared to the Baermann funnel technique, formalin-ether concentration, and direct smear examination was 1.3-, 2-, and 2.6-fold, respectively. The results of the Baermann funnel technique and direct smear examination are in keeping with those of previous studies [22, 34].

We observed that 75% and 34.6% of the culture-positive and ELISA-positive cases were patients treated by steroids, respectively. Immunosuppression caused by corticosteroids can lead to hyperinfection syndrome or disseminated infection in immunocompromised patients [32, 35]. Corticosteroids, by interfering with the Th2 response by binding glucocorticoid receptors in the CD4+ Th2 cells, may change the regular mechanisms of immunity.

The follow-up of positive-culture cases showed recurrent infection in two cases. In one case, the agar plate culture was positive 6 months after the treatment, and in the other case it was positive 8 months after the treatment. Given that ivermectin is currently the treatment of choice for strongyloidiasis [5], it is possible that unavailability of ivermectin in the studied province as well as treatment with albendazole is one of the causes of the recurrence of infection in patients.

This study had limitations that should be considered. First, only one stool sample was examined from each patient. Second, ivermectin was not available in the region, and patients were treated with albendazole. Third, due to the limited budget, PCR was performed only on culture-positive samples. Fourth, data on the CD4 and viral load of the HIV patients and the duration of treatment with corticosteroids were not available in all patients.

## Conclusions

Our findings stress the importance of screening for strongyloidiasis in high-risk patients, particularly in endemic areas. Given that the ELISA test had the highest sensitivity, the screening of all high-risk patients for *S. stercoralis* infection is recommended prior to starting the therapy using the ELISA test. Considering the fact that in two cases *S. stercoralis* larvae were observed 6 months and 8 months after the treatment with albendazole, monitoring of patients is recommended. In addition, ivermectin should be available to strongyloidiasis patients in the endemic areas. Although this study was not focused on patients with eosinophilia, the results indicated the importance of paying attention to patients with unknown eosinophilia in endemic areas.

## Data Availability

Not applicable.

## Abbreviations

NTDs: Neglected tropical diseases
COPD: Chronic obstructive pulmonary disease
PCR: Polymerase chain reaction
STH: Soil-transmitted helminthiasis
HTLV-1: Human T-cell lymphotropic virus type 1
HIV: Human immunodeficiency virus
DM: Diabetes mellitus
ELISA: Enzyme-linked immunosorbent assay
*SSU*: Small subunit (18S) ribosomal DNA
ROC: Receiver-operating characteristic
PPV: Positive predictive value
PNV: Negative predictive value
CBC: Complete blood count
AUC: Area under the curve
